# Mesenchymal Stem Cells Do Not Prevent Antibody Responses against Human α-L-Iduronidase when Used to Treat Mucopolysaccharidosis Type I

**DOI:** 10.1371/journal.pone.0092420

**Published:** 2014-03-18

**Authors:** Priscila Keiko Matsumoto Martin, Roberta Sessa Stilhano, Vivian Yochiko Samoto, Christina Maeda Takiya, Giovani Bravin Peres, Yara Maria Correa da Silva Michelacci, Flavia Helena da Silva, Vanessa Gonçalves Pereira, Vânia D'Almeida, Fabio Luiz Navarro Marques, Andreia Hanada Otake, Roger Chammas, Sang Won Han

**Affiliations:** 1 Research Center for Gene Therapy, Federal University of São Paulo, São Paulo, Brazil; 2 Department of Biophysics, Federal University of São Paulo, São Paulo, Brazil; 3 Carlos Chagas Filho Institute of Biophysics, Federal University of Rio de Janeiro, Rio de Janeiro, Brazil; 4 Department of Biochemistry, Federal University of São Paulo, São Paulo, Brazil; 5 Department of Genetics, Federal University of Rio Grande do Sul, São Paulo, Brazil; 6 Department of Pediatrics, Federal University of São Paulo, São Paulo, Brazil; 7 Nuclear Medicine Center, University of São Paulo, São Paulo, Brazil; 8 Laboratory of Experimental Oncology, Department of Radiology and Oncology, School of Medicine, São Paulo University, São Paulo, Brazil; 9 Translational Investigation Center of Oncology, Cancer Institute of São Paulo State, São Paulo, Brazil; University of São Paulo, Brazil

## Abstract

Mucopolysaccharidosis type I (MPSI) is an autosomal recessive disease that leads to systemic lysosomal storage, which is caused by the absence of α-L-iduronidase (IDUA). Enzyme replacement therapy is recognized as the best therapeutic option for MPSI; however, high titers of anti-IDUA antibody have frequently been observed. Due to the immunosuppressant properties of MSC, we hypothesized that MSC modified with the IDUA gene would be able to produce IDUA for a long period of time. Sleeping Beauty transposon vectors were used to modify MSC because these are basically less-immunogenic plasmids. For cell transplantation, 4×10^6^ MSC-KO-IDUA cells (MSC from KO mice modified with IDUA) were injected into the peritoneum of KO-mice three times over intervals of more than one month. The total IDUA activities from MSC-KO-IDUA before cell transplantation were 9.6, 120 and 179 U for the first, second and third injections, respectively. Only after the second cell transplantation, more than one unit of IDUA activity was detected in the blood of 3 mice for 2 days. After the third cell transplantation, a high titer of anti-IDUA antibody was detected in all of the treated mice. Anti-IDUA antibody response was also detected in C57Bl/6 mice treated with MSC-WT-IDUA. The antibody titers were high and comparable to mice that were immunized by electroporation. MSC-transplanted mice had high levels of TNF-alpha and infiltrates in the renal glomeruli. The spreading of the transplanted MSC into the peritoneum of other organs was confirmed after injection of ^111^In-labeled MSC. In conclusion, the antibody response against IDUA could not be avoided by MSC. On the contrary, these cells worked as an adjuvant that favored IDUA immunization. Therefore, the humoral immunosuppressant property of MSC is questionable and indicates the danger of using MSC as a source for the production of exogenous proteins to treat monogenic diseases.

## Introduction

Mucopolysaccharidosis type I (MPSI) is an autosomal recessive disease that leads to systemic lysosomal storage caused by the absence of the enzyme alpha-L-iduronidase (IDUA) [1], [2]. IDUA participates in the degradation of glycosaminoglycans (GAG), and its absence causes the accumulation of heparan sulfate and dermatan sulfate in various tissues and organs, which causes coarse facial features, mental retardation, skeletal abnormalities, short stature and excess GAG in the urine [3].

Currently, with the high production capacity of the recombinant IDUA enzyme, enzyme replacement therapy (ERT) has become the best therapeutic option for MPSI. Although the cost of treatment is very expensive (US$ 150–300 thousand per year), patients treated weekly with this enzyme via intravenous infusion have shown great improvement. Dramatic reduction in urinary GAG excretion, normalization of hepatosplenomegaly and improved respiratory function and physical capacity were the main benefits that were observed in most patients treated by ERT [4], [5].

The IDUA in circulation is taken up by cells via mannose-6-phosphate receptor through a mechanism known as cross-correction. For efficient ERT, it is essential to maintain the active catalytic site of the enzyme and that these enzymes penetrate efficiently into deficient cells. Despite the existence of this transport mechanism for IDUA, most MPSI patient cells have never interacted with this enzyme. Therefore, IDUA becomes a foreign body that can generate an immune response. In clinical studies of lysosomal storage diseases (LSD) by ERT, alloantibodies were generated in all LSD [4], [5]. The initial clinical studies of ERT for MPSI reported that approximately 40% of patients generated specific antibodies against IDUA, but immunoprecipitation of the enzyme or inhibition of its catalytic activity were not observed [6]. However, a posterior, multinational prospective study showed that patients with a high-titer antibody response showed sub-optimal therapeutic effects when compared to patients who did not have this response [7]. In another study, 91% patients were positive for alloantibodies, but the neutralizing effect of these antibodies against IDUA was unknown [8]. The consequences of these immune responses may affect treatment and could lead to rapid disease progression and subsequent early death [5].

Mesenchymal stem cells (MSC) are able to differentiate into osteocytes, chondrocytes, adipocytes and other cells and are capable of proliferation and adhesion to plastic, which facilitates their cultivation and expansion in large quantities [9]. The main sources of MSC are bone marrow and adipose tissue, but it is known that virtually all tissues possess MSC [10]. One of the properties of MSC is their capacity for secreting immunosuppressive molecules such as nitric oxide [11], [12], prostaglandins, indoleamine 2,3-dioxygenase and IL-6 [13].

The immunosuppressant effects of MSC upon T cells, natural killer cells, dendritic cells and macrophages have been widely studied [14], [15], [16], [17]. Although the immunomodulatory activities of MSC upon B cells are still controversial, strong evidence suggests a delay in B cell maturation and antibody production by MSC in mice [18], [19] and human cell culture [20], [21]; however, their interaction *in vivo* is still not well known.

Because the antibody generation against IDUA is a serious problem when treating MPSI patients with ERT and based on the immunosuppressive property of MSCs, we hypothesized that MSCs modified with an IDUA gene can constitutively produce IDUA because the MSCs could decrease or avoid the generation of anti-IDUA antibodies. To test this hypothesis, we modified MSC with a Sleeping Beauty transposon (SB) vector expressing the human IDUA gene to constantly provide IDUA *in vivo* and injected these cells into the peritoneum of IDUA knockout mice and wild-type mice. The production of anti-IDUA antibodies and IDUA were monitored for weeks to evaluate our hypothesis.

In this study, we used the SB system for gene transfer because it is an integrative, non-viral vector and therefore it is expected to bring about long-term gene expression and an immune response against the vector should be minimal because this system is completely void of viral proteins, which can trigger undesired immune reactions.

## Materials and Methods

### Animals

All procedures involving animals were performed with the approval of the Research Ethics Committee of the Federal University of São Paulo, Brazil (Approval number: CEP 1278/07). IDUA knockout mice (KO) [2] were kindly provided by Dr. Elizabeth Neufeld (UCLA, Los Angeles, USA) and were maintained in our animal house by breeding heterozygous animals (HT). Two-month-old KO mice served as sources for MSC culture establishment; four-month-old KO mice were used for *in vivo* experiments; and three-month-old C57BL/6 mice (WT) were purchased from INFAR (National Institute of Pharmacology, São Paulo, Brazil) for MSC culture establishment and *in vivo* experiments.

### Vectors' construction

The cDNA encoding the human IDUA gene was excised from the pTiger vector [22] using HindIII and inserted in the uP [23] or pVAX vectors (Invitrogen San Diego, CA, USA) for uP-IDUA and pVAX-IDUA construction, respectively. The uP vector contains the complete CMV promoter with enhancer sequences, and the pVAX vector only contains the minimum CMV promoter sequence. For pT2-CMVi-IDUA and pT2-IDUA, the expression cassettes were excised from uP-IDUA and pVAX-IDUA, respectively, using NruI and XhoI and then inserted in the EcoRV site of pT2-BH vector (kindly provided by Dr. Perry B. Hackett of the University of Minnesota, USA). The pT2-CAGGS-IDUA vector promotes IDUA expression by the hybrid CAGGS promoter, which was kindly provided by Dr. Elena Aronovich of the University of Minnesota, USA. The pCMV-SB11 and pCMV-ΔDDE vectors express SB transposase or SB transposase without a catalytic domain, respectively. These vectors were kindly provided by Dr. Perry B. Hackett of the University of Minnesota, USA. The pCMV-SB100X vector was kindly provided by Dr. Zsuzsanna Izsvák and Dr. Zoltán Ivics from the Max-Delbrück-Center for Molecular Medicine, Berlin, Germany.

### Mesenchymal stem cells culture and nucleofection

The KO and WT mice were euthanized by cervical dislocation to obtain mesenchymal stem cells (MSC-KO and MSC-WT, respectively) by flushing the bone marrow of the femur and tibias. Technologies, Carlsbad, USA) supplemented with 10% fetal bovine serum (Life Technologies), 2 mM glutamine (Life Technologies), 50 units/ml penicillin and 50 mg/ml streptomycin sulfate (Life Technologies). The osteogenic and adipogenic differentiation were performed based on an established protocol [22]. After five passages, plasmid delivery was carried out by nucleofection with 10 μg total vector (proportion of the shuttle vector and the SB transposase vector were 1∶1 (w/w)) using the human MSC Nucleofector kit (Lonza, Basel, Switzerland) and program U-23.

### MSC transplantation

The KO and WT mice were treated with 80 mg/kg of isosorbide mononitrate by gavage two hours previous to the procedure. MSC-KO and MSC-WT were nucleofected with the pT2-CAGGS-IDUA and pCMV-SB100X vectors (MSC-KO-IDUA and MSC-WT-IDUA, respectively) and were expanded for 15 days. Four million cells were diluted in 4 ml of DMEM and were injected into the peritoneum of each mouse.

### α-L-Iduronidase enzyme assay

In vitro IDUA dosage was performed using a previously described protocol [22] that utilized 4-methylumbelliferyl-α-L-iduronide (Glycosynth, UK) in fluorometric assays. The IDUA activity from plasma was determined using protocols described by Aronovich et al. [24], and enzymatic activity was expressed as nmol of 4-methylumbelliferone that were released per mg tissue protein per hour (U/mg) or per ml plasma per hour (U/ml).

### Measurement of anti-IDUA antibody

The presence of human anti-IDUA antibody was determined using a method described by Di Domenico et al. [25]. Blood samples were collected fifteen days after the third injection, centrifuged at 500×g/5 min and diluted in 0.1% BSA/PBS. Fifty microliters of diluted serum was then used for the Enzyme-linked immunosorbent assay (ELISA) reaction.

### Histological analysis

The tissues were fixed in 4% paraformaldehyde for 48 hours, dehydrated and embedded in paraffin. Sections of 4-μm thickness were obtained and stained with hematoxylin-eosin (HE) to determine the degree of tissue regeneration and the presence of adipocytes and infiltrated cells. Images were obtained using an optical microscope (Olympus BX60) and analyzed digitally [26].

### ^111^In-labeled MSC distribution

The MSC-KO was cultivated in the previously described conditions. Three million cells were incubated with 10 μCi of ^111^In-oxine for 30 minutes at 37°C. ^111^In-labeled MSC were injected intraperitoneally into six 3-month-old WT mice. Two, four and twenty-four hours after injection, these mice were euthanized by cervical dislocation and the tissues were collected and weighed. The ^111^In-oxine level was measured using the 1282 Compugamma program (LKB Wallac, Gaithersburg, Md.), and the radioactivity in each organ was expressed in two ways: by the counts per unit mass and as a percentage of the injected dose. In all cases, the radioactive decay of ^111^In was corrected to the time of injection. Differences in the radioactivity of the measured organs were determined using analysis of variances (ANOVA) at a threshold of p = 0.05 to indicate a statistical significance.

### Cytokines measurement

The GM-CSF, IFNγ, TNFα, IL-2, IL-4, IL-5, IL-10 and IL-12 in treated KO mice serum from MSC-KO-IDUA (n = 3) and non-treated KO mice (n = 2) were measured fifteen days after the third cell injection using a Bio-Plex Pro Mouse Cytokine 8-Plex panel (Bio-rad, Hercules, CA) in Luminex and analyzed using the Bio-Plex Manager 6.0 software (Bio-rad).

### Intramuscular immunization plus Electroporation *in vivo*

Using a 1 ml insulin syringe, 50 μg of plasmid DNA in 50 μl of PBS was delivered into each of the quadriceps muscles of the mice (25 μg pT2-CAGGS-IDUA plus 25 μg pCMV-SB100 or pCMV-ΔDDE per mouse). Immediately after the DNA injection, electroporation was performed using a needle electrode of 0.5 cm needles of 0.5 mm thickness and with a 5 mm distance between them. Three electric pulses (field strength  = 100 V/cm; pulse length  = 50 ms; ECM 830 field generator, BTX Division, Genetronix, San Diego, CA, USA) were delivered at 1 s of intervals [27].

## Results

### Characterization of MSC and *in vitro* IDUA gene expression

**Figure 1:**
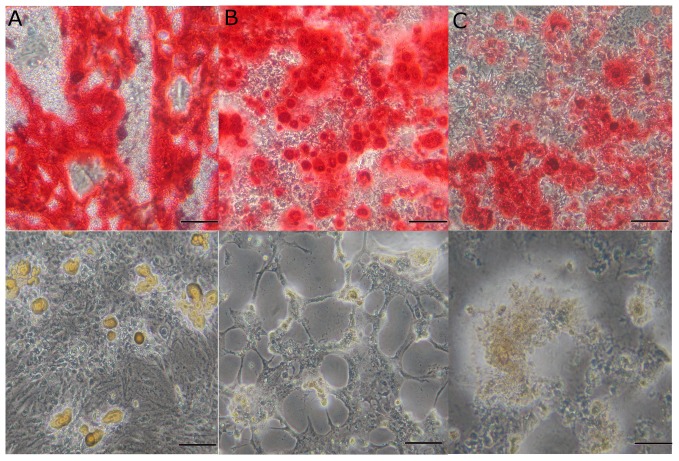
Differentiation and characterization of MSC-KO cultures derived from IDUA-KO mice. MSC-KO cells were differentiated into osteoblasts (upper panel) and adipocytes (lower panel) before (A) and after nucleofection (B, C). Deposits of fat and calcium, which are characteristic of adipocytes and osteoblasts, respectively, are stained in yellow and red, respectively. The original magnification is 100×, and the bars correspond to 50 μm.

MSC were established according to the culture criteria that were described previously. The MSC-KO were maintained in culture for up to 40 passages without morphological changes or differentiation potentials in osteocytes and adipocytes (Figure 1).

**Figure 2:**
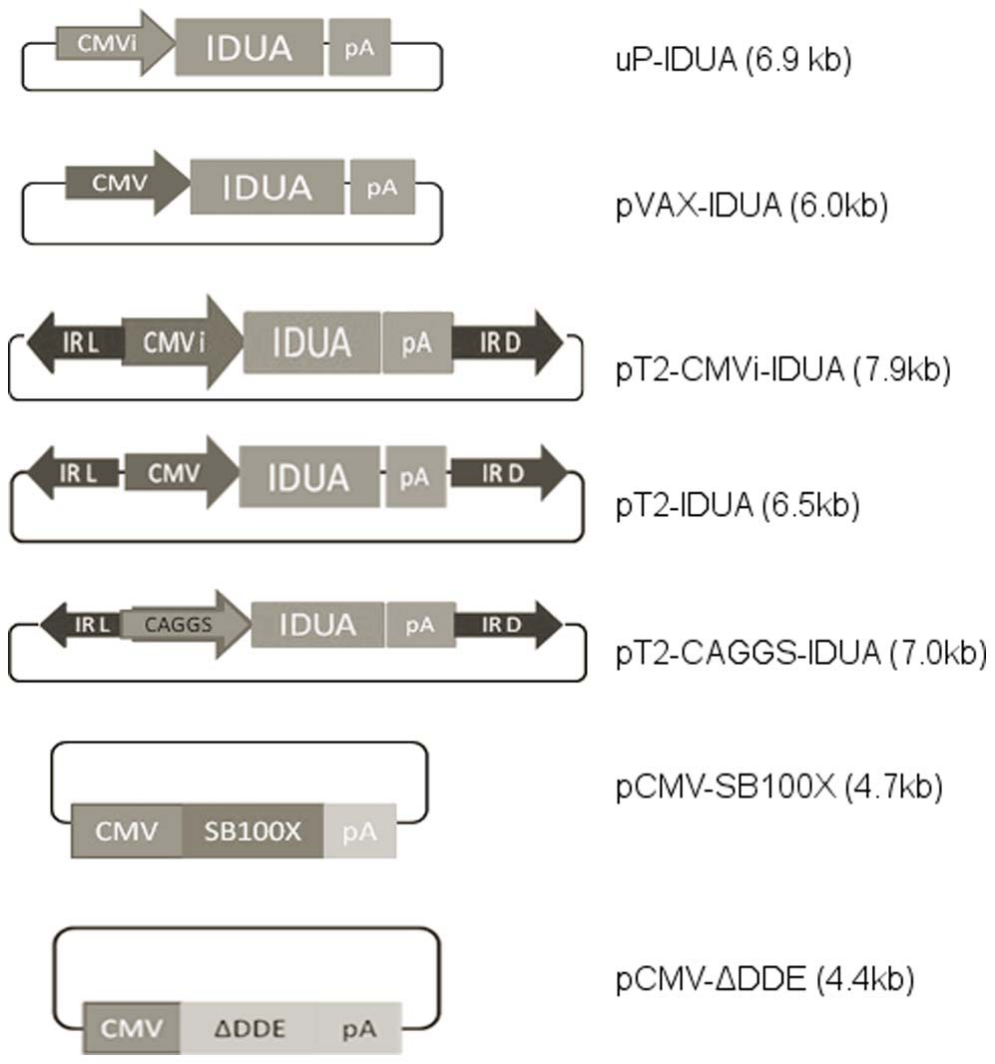
Schematic vector diagrams. CMV: minimum human cytomegalovirus promoter; CMVi: complete human cytomegalovirus promoter; CAGGS: chicken β-actin promoter with CMV enhancer; IDUA: human IDUA cDNA; pA: polyadenylation signal; IR L: left inverted repeated sequence; IR D: right inverted repeated sequence; SB100X: Sleeping Beauty 100X; ΔDDE: Mutated Sleeping Beauty without transposase activity.

**Figure 3:**
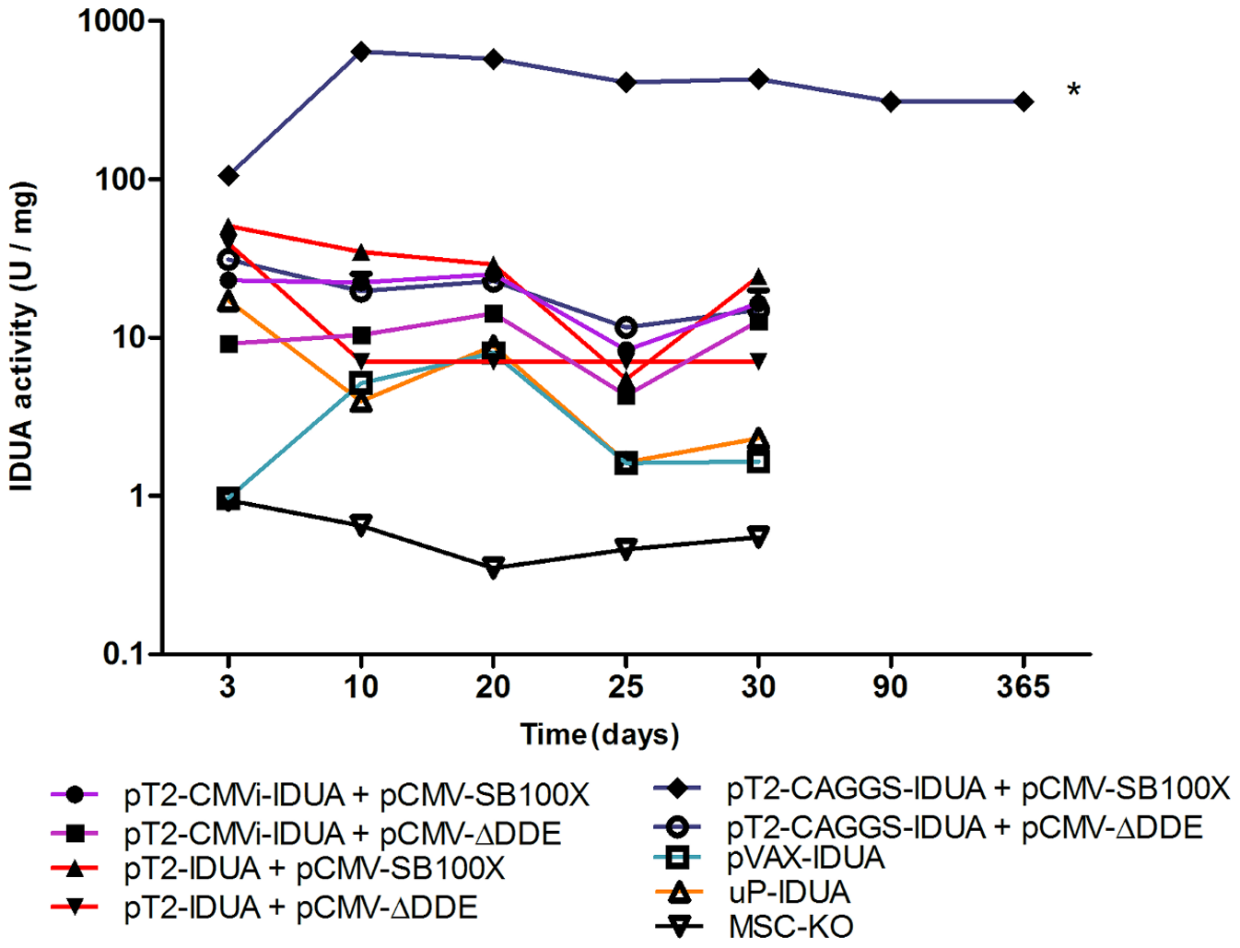
IDUA production by MSC-KO modified with IDUA-expressing vectors. IDUA activity of all nucleofected cells were monitored for 30 days, except for the pT2-CAGGS-IDUA + pCMV-SB100X nucleofected cells, which were monitored for one year. *p<0.0001 for the pT2-CAGGS-IDUA+pCMV-SB100X group compared to other groups. A two-way ANOVA with the Bonferroni post hoc test was used. Vector descriptions are in the Methodology section.

To obtain a large amount of IDUA-producing MSC, the MSC-KO were nucleofected with the following plasmids: pT2-CMVi-IDUA, pT2-IDUA and pT2-CAGGS-IDUA (Figure 2). For vector integration, the MSC were nucleofected with pCMV-SB100X, and pCMV-SBΔDDE (Figure 2) was used as a negative control because this vector did not express the SB transposase. All transfected cells produced IDUA after three days, ranging from 10 U/mg to 100 U/mg (Figure 3), but MSC transfected with pCMV-SB100X and pT2-CAGGS-IDUA maintained the initial level of IDUA throughout the 30-day follow-up (Figure 3). The control without transposase (pT2-CAGGS-IDUA and pCMV-ΔDDE) decreased the expression of IDUA over time because no gene integration occurred. After 3 days, the activity of transfected IDUA cells with pT2-CAGGS-IDUA and pCMV-SB100X quadrupled (105±54 U/mg to 429±98 U/mg). These IDUA-producing cells were frozen. The IDUA activity remained above 300 U/mg for 365 days after nucleofection (Figure 3). Based on these data, MSC modified with the pT2-CAGGS-IDUA and pCMV-SB100X (MSC-KO-IDUA) plasmids were used for therapy in KO mice.

### MSC biodistribution

**Figure 4:**
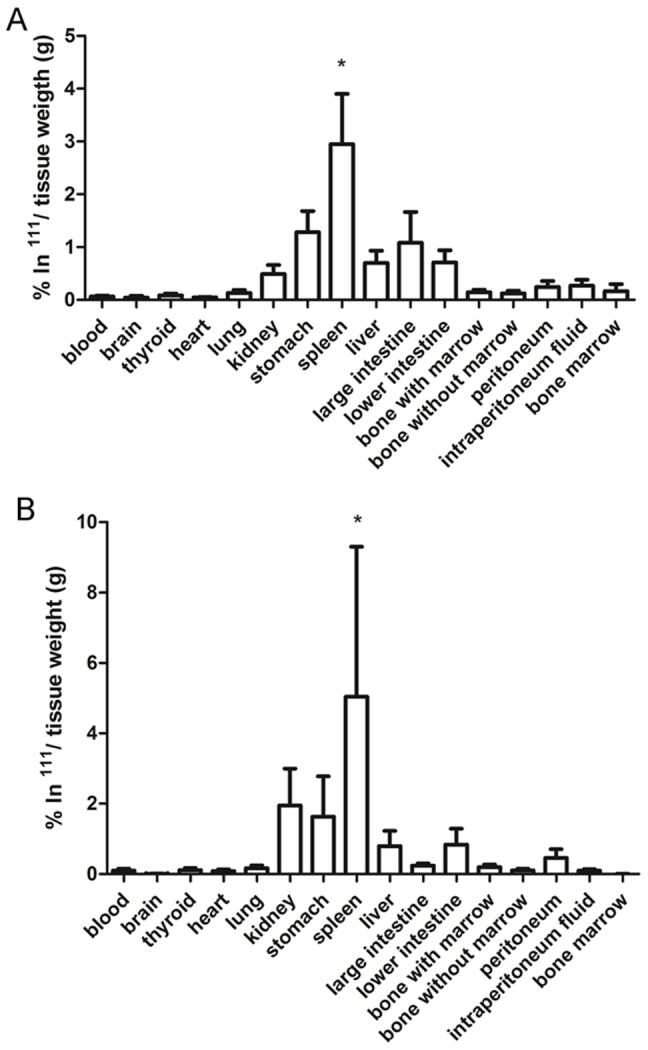
Biodistribution of MSC after injection into the peritoneum. MSC were labeled with ^111^In before injection, and radioactivities in the isolated organs were counted 2 (A) and 24 hours (B) later. Five and three mice were used for 2- and 24-hour experiments, respectively. * p<0.05 when comparing the spleen to all other tissues. A one-way ANOVA with the Bonferroni post hoc test was used.

To determine the biodistribution of the injected MSC, the MSC were radiolabeled with Indium-111 that was conjugated to oxine, injected into the peritoneum of the mice, and the organs were isolated for radioactivity counting. Two hours after MSC injection, radioactivity was detected in the spleen, stomach, large and small intestines, liver and kidney; and 24 hours later, the profile of radioactivity distribution was quite similar to that of 2 hours after injection (Figure 4). The highest radioactivity was found in the spleen and was statistically significant.

### MSC transplantations and antibody responses

**Figure 5:**
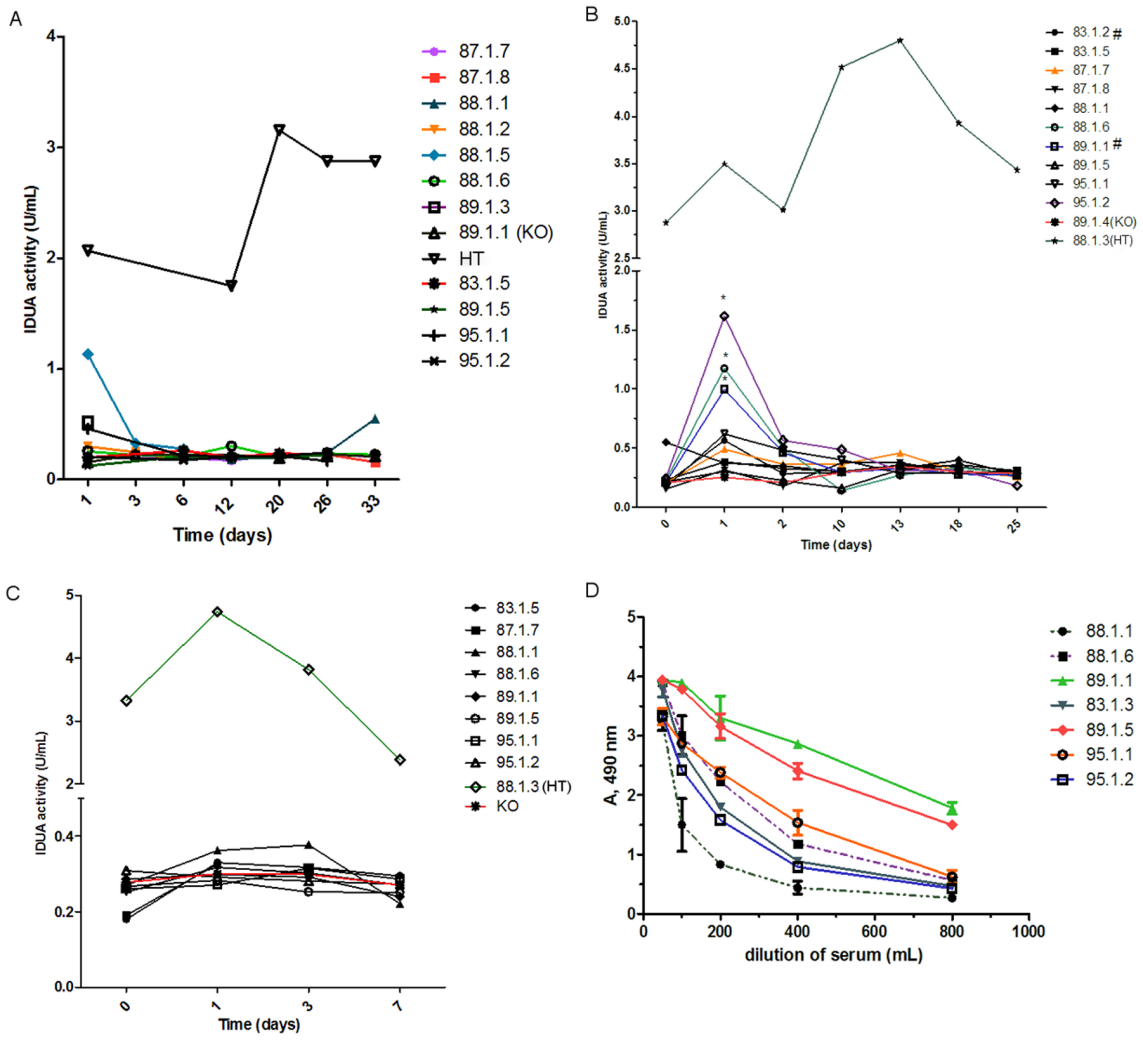
IDUA and anti-IDUA antibody production after MSC-KO-IDUA transplantation. For the first transplantation (A), four million MSC-KO cells were nucleofected with pCMV-SB11 and pT2-CAGGS-IDUA and transplanted into the peritoneum. Three mice died during this experiment: 88.1.2, 88.1.5 and 89.1.3. Approximately, 40 days after the first cell transplantation, the second transplantation was performed using MSC-KO that were nucleofected with pCMV-SB100X and pT2-CAGGS-IDUA (B). Here, two new IDUA-KO mice were included (#). A month after the second transplantation, a third transplantation was performed using the same procedure as the second (C). Fifteen days after the last cell transplantation, blood samples were collected to quantify the anti-IDUA antibody (D). *p<0.05 when comparing the IDUA-KO group to other groups. A two-way ANOVA with the Bonferroni post hoc test was used. HT: heterozygous mouse. KO: IDUA-KO mouse.

For the first MSC-KO implantation, the cells were nucleofected with pT2-CAGGS-IDUA and pCMV-SB11, and 4×10^6^ of these cells that were suspended in 4 ml were injected into the peritoneum of 4-month-old KO mice. These mice produced 28.8±58.7 U/mg of IDUA per mouse, and the final volumes and protein concentrations of the crude extracts were usually 1 ml and 3 mg/ml, respectively; therefore, at the moment of cell injection, these cells were producing approximately 9.6 U of IDUA. Taking into account that a mouse weighing 25 g contains approximately 2 ml of blood, the initial IDUA activity of the MSC-transplanted mouse should be approximately 4.8 U, which corresponds to the activity of a wild-type mouse [22]. Therefore, if this cell transplantation worked as expected, the IDUA activity in the blood would be measurable and the KO mice could be treated. However, after more than a month of follow-up, IDUA activity was not observed in any mouse (Figure 5A). During this experiment, three mice died during the cell transplantation and blood sampling.

One month after the first cell transplantation, these animals were treated again with 4×10^6^ MSC that were modified with SB100X, which produced 359.22±108.16 U/mg. Based on the same calculation as before, we conclude that 120 U was injected into the peritoneum, and this value was 12-fold higher than what was used in the first transplantation. In this experiment, two new KO mice were included for comparison with the other ongoing mice. One day after cell transplantation, more than 1 unit of IDUA activity was present in the blood of the three mice, which represented about a half of the activity of heterozygous mice (Figure 5B). Two more treated mice had slightly elevated IDUA activities, but these values were not statistically significant. However, these IDUA activities decayed soon later, and only two of them had some activity on the 10^th^ day. In the three mice that had higher IDUA activity, one had received MSC-KO-IDUA for the first time, and another new mouse had IDUA activity but its level was low. These results indicated that the transplanted cells could not adapt in the peritoneum and died soon after injection, or the IDUA produced by the transplanted cells were quickly captured by host cells, or the IDUA was neutralized by antibodies. In addition, the first cell transplantation apparently did not cause immunization or tolerance. After the second cell transplantation, two more mice died because of MPSI disease evolution.

When these mice reached approximately 6-months-old, the third injection of MSC-KO-IDUA was carried out with the intention of reverting, at least partially, the disease progression. At this time, we injected the same number of cells, but they produced more IDUA activity: 530.66±59.72 U/mg, which represents an injection of 179 U. The six-month-old KO mice were used to be weakened because of disease progression; consequently, any treatment in this stage was a challenge. After a week of follow-up, we did not detect any IDUA activity in these mice (Figure 5C). However, surprisingly, these mice presented a high titer of anti-IDUA antibody (Figure 5D), which was observed after 15 days of the third cell transplantation.

**Figure 6:**
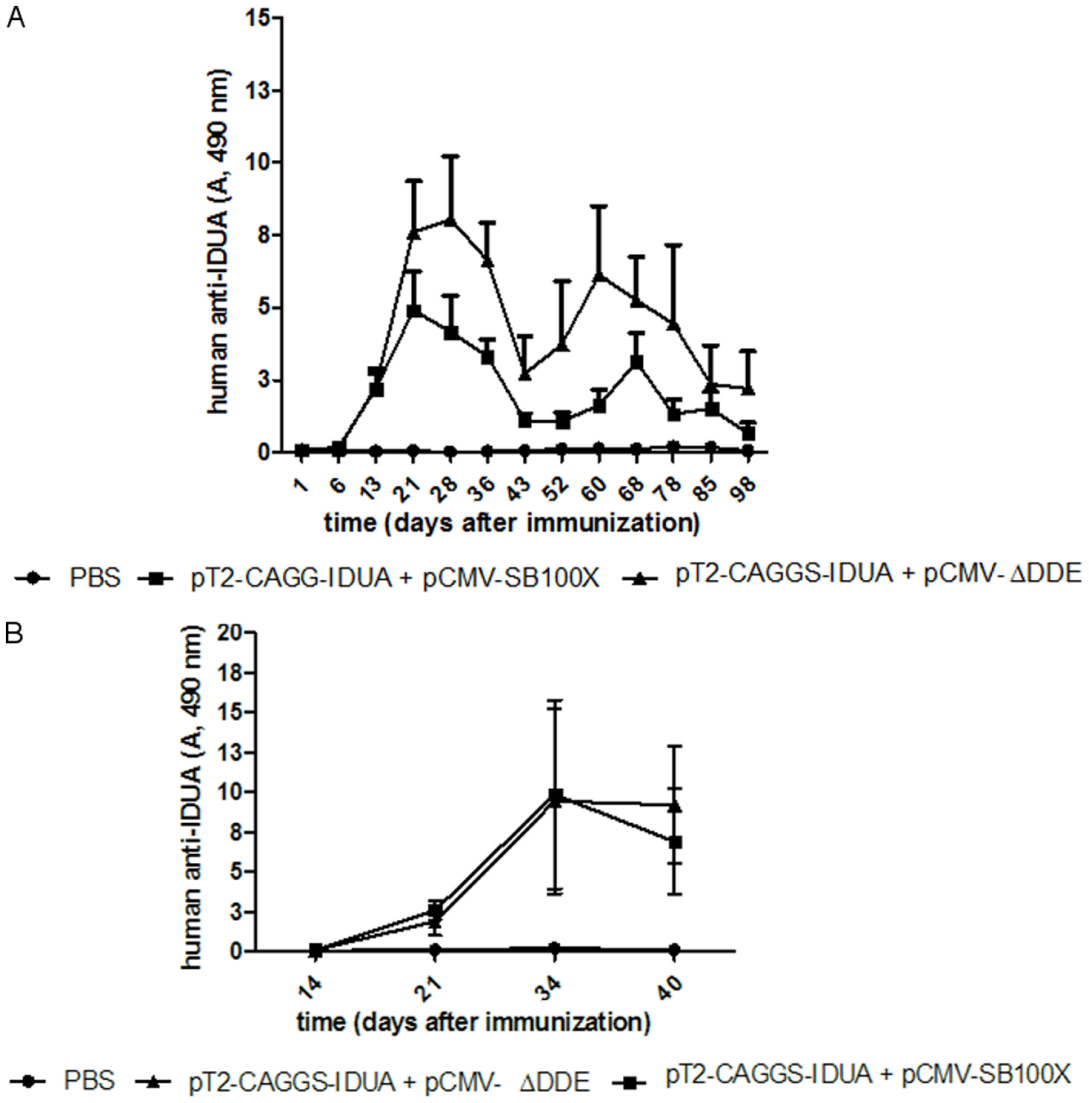
Anti-IDUA antibody production after MSC-IDUA transplantation or electroporation with plasmid vectors. MSC from wild-type mice (C57/Bl6) were nucleofected with pCMV-SB100X and pT2-CAGGS-IDUA and transplanted into the peritoneum of wild-type mice (n = 6 per group) following the same procedure that was used for the KO mice (A). In the negative control group, pT2-CAGGS-IDUA and pCMV-ΔDDE were used. For DNA immunization, pT2-CAGGS-IDUA with pCMV-SB100X or with pCMV-ΔDDE vectors were injected into the thighs of the mice and underwent electroporation (n = 5 per group). A two-way ANOVA with Bonferroni post hoc test was used.

To evaluate the antibody response that was generated against IDUA in the MSC-KO, the same cell transplantation procedure was performed using a MSC-WT that was modified with the same vectors to express IDUA. In this step, we used the MSC-WT to eliminate any interference from the IDUA mutation in the immunosuppressant property of MSC. The MSC-WT-IDUA produced 461.55±52.05 U/mg, which corresponded to 154 U before injection into the peritoneum. Therefore, these values were very similar of those that were used in the previous MSC-KO-IDUA transplantation. After two weeks of cell transplantation, anti-IDUA antibody was detected in all mice and was present until the last assay, which occurred on the 98^th^ day (Figure 6A). The antibody titer of the mice transplanted with MSC-WT-IDUA without SB was only half of that from mice transplanted with MSC-WT-IDUA with SB, which indicated that long-term antigen expression induced a stronger antibody response. Antibody titer decays occurred on the 43^rd^ and 52^nd^ days for unknown reasons, but these levels later returned to normal in both groups. These results clearly demonstrate that MSC did not suppress the antibody response against IDUA.

To understand the immunogenicity of IDUA, WT mice were transfected with the same vectors by electroporation. Electroporation was adopted here because this method brought about better immunization [27]. The antibody response began about a week later than that of the MSC-IDUA transplantation (Figure 6B), but the antibody titer was similar (OD 490 nm≈10), and this level was maintained until the 40^th^ day, which was the day of the last antibody titration.

**Figure 7:**
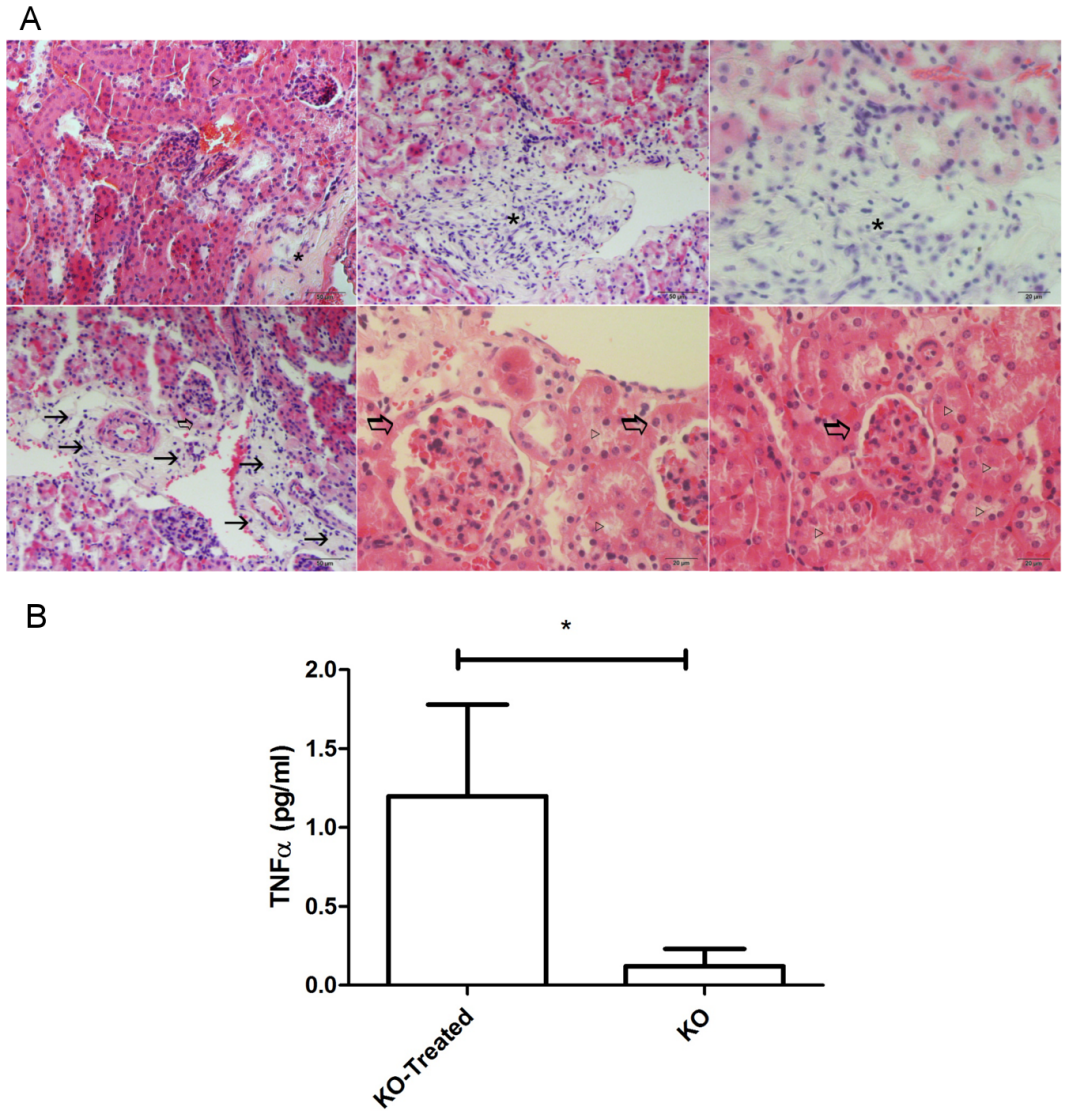
Postmortem analyses of IDUA-KO mice treated with MSC-KO-IDUA by histology and cytokine production. Kidneys from three treated mice were stained with Hematoxylin-Eosin (A). Inflammatory infiltrate in the cortex (*), replacement of normal tissue by inflammatory infiltrate in the renal medulla (→), thickening of the Bowman's capsule (???) and reduction of the lumen of the renal tubules (▹) were marked in the figure. Cytokine production was then evaluated using blood samples from the three treated and two non-treated mice 15 days after the last cell transplantation (B). Only TNF-alpha was detected in the treated mice. *p<0.0001 by the paired student t-test.

As the final experiments, the MSC- KO- IDUA treated mice were killed and their organs were analyzed by histology. Among the changes observed, we found inflammatory infiltrate in the renal glomeruli, thickening of the Bowman's capsule, reduction of the lumen of the renal tubules and replacement of normal tissue by inflammatory infiltrate in the renal medulla (Figure 7A). These histological alterations are typically observed in kidneys during the filtration of immune complexes, which are formed by antigens and antibodies. Therefore, this evidence also supports the previous results that indicate that antibody responses were increased by MSC-KO-IDUA or MSC-WT-IDUA transplantations.

The cytokine profiles of MSC-KO-IDUA-treated mice were then analyzed using blood samples that were collected 15 days after the last cell transplantation, and the non-treated WT mice were used as a control. Among the analyzed cytokines, only TNF-alpha was present in MSC-KO-IDUA-treated mice and was 5-fold higher than that of the control group (Figure 7B), which indicated a state of inflammation in these mice.

## Discussion

Monogenic diseases are of great interest for gene therapy studies because these diseases currently have no effective treatment. MPSI patients must have a constant supply of IDUA to relieve disease manifestation, and this can be performed by enzyme replacement therapy. It has been observed that anti-IDUA antibodies can be generated by ERT [4], [5], [6], [7], [28], [29], and such an antibody response is somewhat expected because the IDUA is a new protein in these patients. However, in animal models, it was shown that ERT or gene therapy started at birth could overcome partially antibody response and improve outcome in difficult-to-treat organs [30], [31], [32]. Based on the immunosuppressant properties of MSC [11], [12], [13], [14], [17], [20], [21], we hypothesized that the production of IDUA by these cells could avoid the antibody response and become a long-term IDUA producer.

The SB system has been proven to be efficient to gene transfer and long-term gene expression in many mammalian cells, including MSC [33]. Here, we showed that this system is also efficient in modifying MSC-KO by nucleofection (Figure 2) because IDUA gene expression has remained nearly constant for a year with the same level as observed initially. The increased IDUA activity during the first week was likely due to continuing integration activity by the SB100X transposase, which led to multiple gene copy integrations per cell [33] and/or by high SB100X activity during the first week after nucleofection [34]. In addition, the property of differentiating into osteocytes and adipocytes (Figure 1) was maintained after transfection, which is an important characteristic of these cells [9], [10], [35].

Because MPSI is a monogenic disease that affects all patient cells, an ideal therapy should be one that could provide IDUA to all affected cells, but this practice is not feasible at this moment. The peritoneum is a highly vascularized region that is easy to access; therefore, a large volume of MSC can be injected. These injected cells can rapidly traffic to affected organs, such as the liver, spleen and kidneys, because of its proximity to these organs. In addition, secreted IDUA will circulate easily and provide this enzyme to the body. The ability for MSC trafficking through the peritoneum is not clearly known, but because macrophages are located in this space and they can traffic through other organs [36], [37], it is expected these cells have a similar mobility. To monitor the mobility of MSC through the peritoneum, radiolabeled MSC were injected into the peritoneum, and the organs were removed later to quantify radioactivity. The vessel dilator isosorbide mononitrate was administered before cell injection to enhance cell trafficking through the peritoneum. White-blood-cell labeling with ^111^In-oxine is a well-known procedure for analyzing infection and inflammation in animals and humans [38], [39]. Because ^111^In-oxine is a neutral molecule, it can easily penetrate the cell membrane, thus allowing ^111^In to attach to an intracellular component such as lactoferrin [40] and causing 8-hydroxyquinoline to be released by the cell. The exchange of 8-hydroxyquinoline by an intracellular component only occurs if the intracellular component can form a more stable complex; therefore, it is expected that the labeled MSCs can hold ^111^In until their death. Because our biodistribution experiments lasted only 24 hours, we expected that only a minimum number of MSC would die. In addition, because the half-life of this radioactivity is 2.8 days, counting the radioactivity 24 hours after injection will provide reliable radioactivity counting because the remaining activity at this time would be approximately 80% of the initial value. Under these experimental conditions, we observed that the spleen was the main organ that received the injected MSC. However, the stomach, kidneys and intestine, which are adjacent to the peritoneum, also had high radioactivity (Figures 4A and 4B). In addition, the basal radioactivity found from the peritoneum and the intraperitoneum fluid 2 hours after injection indicates high mobility of MSC from the peritoneum to circulation.

These data indicate that the distribution of the MSC-KO-IDUA through the body by intraperitoneal injection was an easy and productive procedure.

To test the hypothesis that MSC modified with IDUA could become a good, long-term source of IDUA in vivo because of the immunosuppressant properties of MSC, four million MSC-KO-IDUA were injected into the peritoneum three times over one-month intervals. In an attempt to minimize the immune response, MSC were modified with SB11 to produce a low level of IDUA and were used in the first injection, and for the second and third injections, they were modified with SB100X to produce high levels of IDUA. Considering that the total blood volume of a mouse weighing 25 g is approximately 2 ml, the expected IDUA activities in the blood after injections are 4.8, 60 and 90 U/ml for the first, second and third injections, respectively. Therefore, the units produced by the first injection are similar to that produced by a wild-type mouse (4±2.5 U/ml) [24], [31], [41], and the units produced by the other injections are well above this level and are comparable to human patient submitted to ERT [6], [28]. However, no enzyme activity was detected after the first injection and only a small peak of IDUA production (less than 2 U/ml) was observed one day after the second injection, but this level was not sustained days later (Figures 5A and 5B). To verify the low production of IDUA after MSC-KO-IDUA administration, a third injection was carried out using MSC-KO-IDUA, which produced high IDUA activity; however, no enzymatic activity was detected in any of the mice (Figure 5C). Unlike IDUA activity, these MSC-KO-IDUA-treated mice presented high titers of anti-IDUA antibody (Figure 4D), high concentration of TNF-alfa (Figure 7B) and damaged cortical and medullar kidney tissue (Figure 7A). These results led us to doubt that the immunosuppressant property of MSC from KO mice could be lost or reduced due to IDUA gene mutation or GAG accumulation in MSC. To better investigate this question, MSC-WT was transfected with IDUA and injected in WT mice following the same protocol that was used in the KO mice. In this experiment, no IDUA was detected, but the human anti-IDUA antibody was detected on the 13^th^ day after MSC injection and reached its highest titer on the 21^st^ day (Figure 6A). This antibody response was faster than the classic DNA immunization by electroporation [27] that was at the 21^st^ day and reached its highest point on the 34^th^ day after immunization with a similar optical density (Figure 5B). Taking into consideration that the human and murine IDUA have approximately 80% protein homology, it was surprising to have an antibody response within two weeks that lasted more than 100 days in some animals after only a single injection (Figure 6A). This experiment does not provide information about the preservation of the immunosuppressant property of MSC-KO; however, it clearly shows that these MSC do not suppress human anti-IDUA antibody generation, as was expected. In addition, the antibody responses that were generated after in vivo transfection of WT mice with IDUA vectors by electroporation, which produced a similar level of mice that were transplanted with MSC-WT-IDUA or MSC-KO-IDUA, indicated that the participation of MSC in immunosuppression should be minimum.

The immunosuppressant activity of MSC in vivo seems to be controversial. For example, the treatment of the autoimmune disease Systemic Lupus Erythematosus (SLE) with bone marrow MSC increased the survival and decreased the level of circulating anti-dsDNA [42]. A similar result was observed in a NZBxNZW F1 mouse model that was treated with MSC from adipose tissue for disease prevention. However, the therapeutic effect was lost when the treatment began after disease onset [43], the survival time did not increase, and the formation of the anti-dsDNA antibody could not be avoided [44]. Therefore, more in vivo experiments will be necessary to better define the immunosuppressant or proinflammatory role of MSC.

In conclusion, our in vivo study with MSC modified to constitutively produce IDUA showed an unexpected adjuvant effect of MSC for immunization, which raised high titers of an anti-IDUA antibody. This antibody response was as strong as DNA immunization by electroporation and lasted longer. Therefore, the use of genetically modified MSC for the long-term production of IDUA in KO mice to treat MPSI still faces unavoidable antibody responses. Our studies have been carried out in a murine model using the human IDUA gene, but the use of human MSC as a source for production of exogenous proteins to treat monogenic diseases must be well validated before it is clinically applied.
